# Treadmill exercise ameliorates the regulation of energy metabolism in skeletal muscle of NSE/PS2mtransgenic mice with Alzheimer’s disease

**DOI:** 10.20463/jenb.2017.0046

**Published:** 2017-03-31

**Authors:** Jang-Soo Yook, Joon-Yong Cho

**Affiliations:** 1 Laboratory of Exercise Biochemistry and Neuroendocrinology, Faculty of Health and Sports Sciences, University of Tsukuba, Tsukuba Japan; 2 Department of Exercise Biochemistry, Korea National Sport University, Seoul Republic of Korea

**Keywords:** Alzheimer’s disease, Skeletal muscle, Treadmill exercise, β-amyloid

## Abstract

**[Purpose]:**

Alzheimer’s disease (AD) is classified as a progressive neurological disorder, which not only causes cognitive impairment but also abnormal weight loss, with a reduction of muscle mass related to the accumulation of amyloid-β (Aβ) in skeletal muscle. Thus, we investigated the effect of treadmill exercise on Aβ deposition, and p-AMPK, p-ACC, BDNF, and GLUT4 protein levels the regulation of muscle energy metabolism using an AD mouse.

**[Methods]:**

At 13 months of age, NSE/PS2m mice (Tg) and control mice (non-Tg) were assigned to non-exercise control (Con) and exercise groups (Exe). The four groups were as follows: non-Tg Con, non-Tg Exe, Tg Con, and Tg Exe. The treadmill exercise was carried out for 12 weeks.

**[Results]:**

The highest levels of Aβ expression in the skeletal muscle were in the Tg Con group. Aβ expression was significantly reduced in the Tg Exe group, compared to the Tg Con group. Congo red staining showed remarkable diffuse red amyloid deposition in the Tg Con group, while Aβ-deposition in the skeletal was reduced with muscle exercise in the Tg Exe group. Exercise also increased AMPK and ACC phosphorylation and BDNF and GLUT4 expression in the skeletal muscle of non-Tg and Tg mice.

**[Conclusion]:**

Treadmill exercise reduces Aβ-deposition in the skeletal muscle and improves the regulation of energy metabolism. Thus, collectively, these results suggest that exercise could be a positive therapeutic strategy for skeletal muscle dysfunction in AD patients.

## INTRODUCTION

The loss of brain cells in Alzheimer’s disease (AD), which is classified as a progressive neurological disorder, causes irreversible deterioration of cognitive function and loss of memory. Although the pathogenic mechanism of AD remains unclear, the primary pathological hallmarks of AD are extracellular senile plaques that are composed of amyloid-β (Aβ), and intracellular neurofibrillary tangles of abnormal fibers, which lead to neuronal death^1^. Many previous studies have reported that the accumulation of abnormal Aβ, which is cleaved by β-and γ-secretases derived from the amyloid precursor protein (APP), provokes an outstanding increase of the neurotoxic Aβ in the brain, leading to cognitive impairments^2,3^. Therefore, most common therapies that are currently being researched to prevent and treat AD are focused on reducing the level of Aβ deposition in the brain, in order to improve cognitive function. 

Although the effects of Aβ accumulation on brain function have been widely investigated, very little is known about its harmful effects on non-neuronal tissue, including skeletal muscle. Previous studies have reported that an elevation of Aβ deposition in skeletal muscle was observed in both AD patients, and a transgenic animal model of AD with a *PS2m* gene mutation^4,5^. In particular, the clinical symptoms of AD patients include lifestyle difficulties such as general weakness, weight loss, and fatigue^6-8^. Accumulated Aβ in skeletal muscle contributes to motor dysfunction through muscle weakness and atrophy, which has been observed in AD as well as other disorders with associated gait disturbances^9,10^. According to these studies, the adverse effects of Aβ deposition may not only manifest in the brain, but also in skeletal muscle. 

A number of molecular mechanisms have also been identified with respect to cell signaling in AD. Several studies have focused on the correlation between cellular energy homeostasis and the risk of AD^11^. AMP-activated protein kinase (AMPK) plays an important role in the regulation of energy homeostasis of the entire body^12,13^. Interestingly, a number of studies have implied that AD is associated with abnormal energy metabolism in the brain, including mitochondrial dysfunction and the decline of glucose uptake, which is potentially related to AMPK dysregulation^14-16^. Moreover, AMPK activation stimulates the GLUT4 glucose transporter, which regulates glucose metabolism following adaptation to endurance exercise in skeletal muscle^17-19^. During lipid metabolism, the activation of AMPK in skeletal muscle results in the increased phosphorylation of acetyl coenzyme A carboxylase (ACC), which in turn catalyzes the biosynthesis of fatty acids^20,21^ and is inactivated in response to exercise^22,23^. 

Brain-derived neurotropic factor (BDNF) is a member of the neurotrophin family. BDNF plays a role in neuronal survival and growth, and synaptic function^24^. A reduction in BDNF influences neurodegenerative diseases such as AD and Parkinson’s disease, where it is associated with a dysfunction in learning and memory^25,26^. Interestingly, previous studies have also shown that BDNF is expressed in skeletal muscle, where its levels increase in response to exercise via the phosphorylation of AMPK and ACC^27^. The inactivation of AMPK has recently been linked to specific metabolic and neurodegenerative diseases, including AD and diabetes^28,29^. Studies on ADs have reported that the activation of AMPK can reduce the production of Aβ in neurons^30,31^. Therefore, AMPK activation may lead to beneficial effects on energy metabolism, not only in the brain, but also in skeletal muscle in individuals with AD. 

Moreover, there is evidence to suggest that AMPK activation has a beneficial impact on curing and reducing the risk of AD pathogenesis. Furthermore, several studies have specifically reported that chronic exercise plays an important role in enhancing the physical strength in older individuals^32,33^ and facilitating the improvement of cognitive function^34,35^. Previous studies on humans suggest that exercise and physical activity improves overall health and quality of life, prevents complications, and delays the onset of AD^36,37^. In addition, previous biological research conducted using animal models of AD has demonstrated that voluntary and forced exercise may decrease risk factors for AD and prevent memory deficits, through the reduction of Aβ accumulation and promotion of neuronal plasticity^5,38-41^. Taken together, these findings suggest that exercise has therapeutic potential for AD by reducing neuronal cell death. Considering the type of regular exercise, skeletal muscle dysfunction in AD may also be restored through exercise-induced molecular regulation of energy metabolic pathways. 

In the present study, we investigated whether treadmill exercise might reduce the accumulation of Aβ-42 protein levels and improve several molecular factors, namely, p-AMPK, p-ACC, GLUT4, and BDNF, which are related to energy metabolism in skeletal muscle. To address our hypothesis, we used a transgenic mouse model of AD with skeletal muscle-specific overexpression of the PS2m gene, which is usually confined to the brain region^42^. 

## METHODS

### Animal experimentation

**Figure 1.**
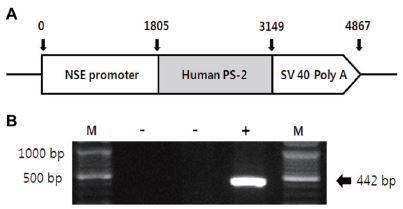
Identification of NSE/hPS-2m mouse gene expression. (A) hPS-2m was placed under the control of the NSE promoter gene. (B) The *PS2m* gene was identified in genomic DNA isolated from the tail using RT-PCR. The 442-bp products were shown in Tg mice carrying only the NSE/hPS-2m mutant gene.

Transgenic (Tg) and non-transgenic littermates (non-Tg) were provided by the Department of Laboratory Animal Resources at the National Institute of Toxicological Research, Korea Food and Drug Administration (KFDA). NSE/PS2m Tg mice expressing the human mutant *PS2* gene under the control of neuron-specific enolase (NSE) promoter^42^ were used in this study, as illustrated in Figure 1A. All mice were aged 13 months, and were separated into either the exercise or sedentary group (*n*=10). Both groups were divided into transgenic and non-transgenic (*n*=10) subgroups according to the genotype of the *PS2m* gene mutation. All mice were kept in cages under a strict 12:12 hour light/dark cycle at 22±2 °C and 50% relative humidity, and were fed a standard chow diet (Purina Mills, Seoul, Korea). The study was conducted under the regulations of an accredited Korea FDA animal facility following the AAALAC International Animal Care Policies (Accredited Unit-Korea FDA: Unit Number-0009966). All pedigrees were hemizygous for each transgene. 

### Treadmill exercise

AD mice were divided into four groups according to the expression of the transgene and the type of treatment. The groups consisted of exercise transgenic (Tg Exe, *n*=5) and non-transgenic mice (non-Tg Exe, *n*=5), and sedentary transgenic (Tg Con, *n*=5) and non-transgenic mice (non-Tg Con, *n*=5). The exercise was carried out with a treadmill apparatus for rodents (Dae-myung Scientific Co. Ltd., Korea). Exercise groups (Tg Exe and non- Tg Exe) were habituated to treadmill running at 5 m/min for 10 min/day for 1 week. The mice ran on the treadmill continuously with no incline for 60 min/day, 5 days/week after the habituation period, as conducted previously^5^. The running time (from 30 to 60 min) and treadmill speed (from 5 to 12 m/min) were gradually increased over 12 weeks. The non-Tg Con and Tg Con groups were not trained with treadmill exercise. 

### Reverse transcriptase-PCR analysis

To identify the expression of the NSE/hPS-2m gene, genomic DNA isolated from the tails of all mice was analyzed to identify the *PS2m* gene by reverse transcriptase (RT)-PCR, as previously described^42^. The *PS2m* gene was synthesized using sense primer (5’-GAGGAAGAAGTGTGT GATGAG-3) and antisense primer (5’-CACGATGACGCTGATCATGATG-3), with a complementary *PS2m* gene ranging from 395 to 416 and from 817 to 796 nucleotides as the DNA template. The reaction mixtures containing PCR PreMix solution (Bioneer, CA, USA) were amplified using a Perkin-Elmer thermal cycler to subject the samples to 25 cycles of the following: denaturation at 94 °C for 30 s, annealing at 62 °C for 30 s, and elongation at 72 °C for 45 s. These primers led to the amplification of a 442-bp segment of the *PS2m* sequence (Fig. 1B). According to this process, all mice were classified as either non-Tg or Tg. 

### Western blot analysis

For western blot analysis, proteins (40 μg) were prepared from the plantaris muscle of the experiment models by the homogenization of tissue. The protein content in each sample was then determined using a BCA assay kit (Pierce biotechnology Inc. Rockford, IL). Proteins from each tissue were applied to a 10% gradient gel of polyacrylamide (SDS-PAGE) by electrophoresis for 3 h, and were transferred to a PVDF membrane (Immuno Blot PVDF membrane, Bio-Rad, CA, USA) with transbuffer for 2 h using a continuous electroblot. The membrane was blocked using a blocking solution at 1:5,000, and subsequently incubated overnight at 4 °C in a blocking buffer with the following primary antibodies, diluted at ratios of 1:1,000 to 1:5,000: anti-AMPKα (Cell Signaling Technology, #2532, MA, USA), anti-phospho-AMPKα (Cell Signaling Technology, #2531, MA, USA), anti-GLUT4 (Abcam, ab654, MA. USA), anti-ACC (Cell Signaling Technology, #3662, MA, USA), anti-phospho-ACC (Cell Signaling Technology, #3661, MA, USA), anti-BDNF (Santa Cruz Biotechnology, sc-546, CA, USA), and anti- Aβ (Cell Signaling Technology, #2454, MA, USA). The membranes were the washed with PBS-T buffer (137 mM NaCl, 2.7 mM KCl, 10 mM Na2HPO, and 2 mM KH_2_PO_4_), including 0.05% Tween-20 (Sigma, MO), several times, before being incubated with the following secondary antibodies (at a dilution of 1:1,000 dilution): HRP goat-anti-rabbit (Invitrogen, 656120, CA, USA) for AMPKα, phospho-AMPKα, GLUT4, ACC, BDNF, and Aβ, and Peroxidase rabbit-anti-goat (Invitrogen, 611620, CA, USA) for phospho-ACC. The membranes were incubated with the appropriate secondary antibody for 1 h at room temperature (RT), before being developed using a western blot analysis system (Santa Cruz Biotechnology, CA, USA). Densitometric scanning was conducted using a ChemiDoc XRS system (Bio-Rad, CA, USA). 

### Congo red staining

For histological staining with Congo red, mice were perfused with 4% paraformaldehyde buffer via the left cardiac ventricle. The plantaris muscle was subsequently removed and fixed in 10% PFA for 12 h on a shaker at RT. The paraformaldehyde-fixed tissue was then quickly washed three times with 70% ethanol, on a shaker, before being incubated in the same for a minimum of 12 h at 4 °C. All tissue was then embedded in paraffin, and sectioned at a thickness of 6 μm. Slides were then deparaffinized, hydrated with descending grades of xylene, and incubated in Congo red solution for 60 min at RT. After incubation, the slides were washed in distilled water and briefly rinsed in an alkaline alcohol solution (1% sodium hydroxide mixed with 50% ethanol). The sections were counterstained with hematoxylin for the detection of Congo red-positive amyloid, and rinsed with ascending grades of 95% ethanol, cleared in xylene, and mounted on coverslips with Permount. 

### Statistical analysis

All data were analyzed using SPSS software (version 10.0, SPSS Inc., Chicago, IL, USA). Values were expressed as mean ± standard deviation (SD). Western blot results were analyzed using a two-way ANOVA followed by Fisher’s LSD *post-hoc* test for multiple comparisons, to identify whether there was a statistically significant interaction or main effect among the groups. All analyses were converted to graphs using GraphPad Prism 5.0. Differences were considered statistically significant at *p* < 0.05. 

## RESULTS

### Effects of treadmill exercise on the level of Aβ-42 in skeletal muscle

**Figure 2.**
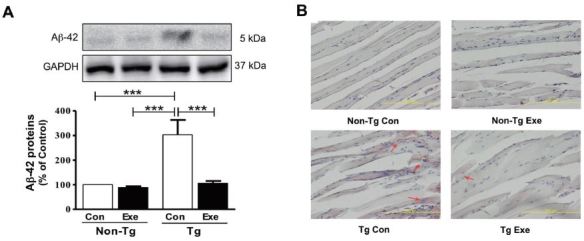
Effects of treadmill exercise on the level of Aβ-42 in skeletal muscle of NSE/PS2m mice. The treadmill exercise reduced the levels of Aβ-42 expression and distribution of red Aβ deposition in the plantaris muscle of PS2-Tg mice. (A) Relative levels of Aβ-42 were analyzed in triplicate by western blot. The band intensity of Aβ-42 expression was normalized to GAPDH levels as an internal control. The values were converted to percentage of the non-Tg Con group (each value represents mean ± SD, *n*=5/group). Data were analyzed using a two-way ANOVA followed by Fisher’s LSD *post-hoc* test (***P < 0.001). (B) Immunostaining of Aβ deposition in the plantaris muscle, which was assayed using Congo red staining, was seen in Tg mice (see red arrows), but not in non-Tg mice.

We previously demonstrated that treadmill exercise reduced the amount of Aβ-42 deposit in the skeletal muscles of NSE/PS2m Tg mice, at 12 months of age^5^. Thus, in the current study, we initially investigated whether 12 weeks of treadmill exercise had beneficial effects on the reduction of Aβ-42 in the skeletal muscle of Tg mice. The results showed that Tg mice that were sedentary showed a higher level of Aβ-42, compared to sedentary non- Tg mice (F_(3,16)_=114.04, *p* < 0.001; Fig. 2A). However, the expression of Aβ-42 in skeletal muscle significantly decreased in Tg mice that were subjected to treadmill exercise, compared to sedentary Tg mice (F_(3,16)_=106.10, *p* < 0.001; Fig. 2A). In addition, histological staining with Congo red was applied to visually identify the distribution of Aβ in the plantaris muscle. Diffuse amyloid deposition, which was stained by Congo red, was only found in Tg mice, but its distribution was markedly reduced in Tg mice that performed 12 weeks of treadmill exercise training (Fig. 2B). Thus, these results suggest that chronic treadmill exercise has positive effects on decreasing the levels of Aβ in skeletal muscle. 

### Effects of treadmill exercise on the expression of AMPK, ACC, BDNF, and GLUT4 in skeletal muscle

**Figure 3.**
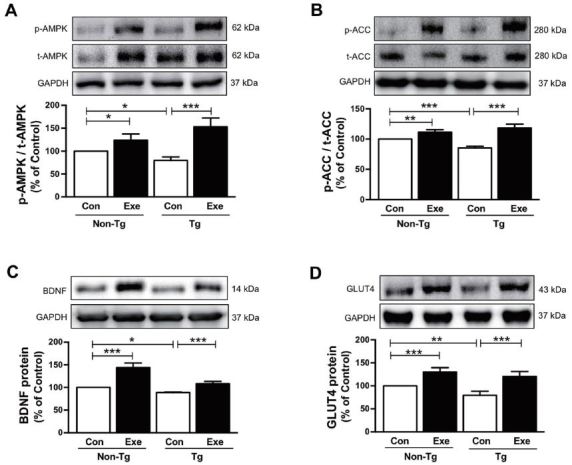
Effects of treadmill exercise on the expression of p-AMPK, p-ACC, GLUT4, and BDNF proteins in skeletal muscle of NSE/PS2m mice. The protein levels of (A) p-AMPK, (B) p-ACC, (C) BDNF, and (D) GLUT4 in the plantaris muscle were significantly decreased in the Tg Con group, relative to the non-Tg group. Compared to sedentary groups, the exercise groups showed a significant increase in the phosphorylation of AMPK and ACC, and in the expression of GLUT4 and BDNF, in the plantaris muscle. Representative protein bands and graphs were assayed in triplicate by western blots. Band intensities were normalized to GAPDH (internal control). The values were converted to percentages of the non-Tg Con group (each value represents mean ± SD, *n*=5/group). The data were analyzed using a two-way ANOVA followed by Fisher’s LSD *post-hoc* test (*P < 0.05, **P < 0.01, ***P < 0.001).

Although Aβ-induced pathogenic mechanisms in skeletal muscle remain unclear, several previous studies have indicated abnormal regulation of glucose and fat metabolism in mouse models of AD that were engineered to specifically accumulate Aβ in skeletal muscle^43-45^. Thus, we examined if treadmill exercise increases the level of AMPK, which is a recognized key regulator of skeletal muscle fat metabolism, and ACC and BDNF, which are known to regulate fatty acid oxidation^27,46^. The results in the current study demonstrated that sedentary Tg mice expressed significantly lower phosphorylation levels of AMPK (F_(3,16)_=14.638, *p* < 0.01; Fig. 3A) and ACC (F_(3,16)_=27.50, *p* < 0.001; Fig. 3B), and lower BDNF expression (F_(3,16)_=66.38, *p* < 0.001; Fig. 3C), in comparison to sedentary non-Tg mice. However, the phosphorylation of AMPK (F_(3,16)_=60.40, *p* < 0.001; Fig. 3A) and ACC (F_(3,16)_=118.17, *p* < 0.001; Fig. 3B) and the expression of BDNF (F_(3,16)_=122.75, *p* < 0.001; Fig. 3C) significantly increased after 12 weeks of treadmill exercise in sedentary non-Tg and Tg mice. In addition, since GLUT4 plays an important role in the regulation of glucose metabolism that is responsible for insulin-regulated glucose uptake in the skeletal muscle47, next we determined if the treadmill exercise affected its level in skeletal muscle of the NSE/PS2m mice. Sedentary Tg mice had a lower GLUT4 expression, than non-Tg Con mice without exercise (F_(3,16)_=12.68, *p* < 0.01; Fig. 3D). However, 12 weeks of treadmill exercise significantly increased the expression of GLUT4 in non-Tg and Tg mice (F_(3,16)_=70.50, *p* < 0.001; Fig. 3D). Taken together, these results suggest that treadmill exercise reverses the Aβ deposition-induced downregulation of AMPK, ACC, BDNF, and GLUT4 expression, which in turn is associated with impairment of energy metabolism in skeletal muscle of AD subjects. 

## DISCUSSION

AD is a common neurodegenerative disorder, typically diagnosed in elderly people, extensively impairs cognitive function. Aβ deposition, which is present in senile plaques, are thought to be neurotoxic, and thus, is suspected of playing a primary role in the pathogenesis of AD^1,48^. Its efficacy as a non-pharmacological treatment for AD has been previously demonstrated in studies showing the effect of chronic exercise on suppressing the induction of Aβ in the brain and ameliorating memory impairment in several mouse models of AD^5,39,40,49^. Additionally, since the accumulation of Aβ, the pathological hallmark of AD, was also observed in skeletal muscle of both human patients and animal models of AD^4,5,45^, we investigated the effect of treadmill exercise on Aβ deposition and energy metabolism in skeletal muscle, using the NSE/PS2m-transgenic mouse model of AD. One of the most important findings of the present study was that treadmill exercise decreased Aβ-42 protein levels and increased p-AMPK, p-ACC, BDNF, and GLUT4 protein levels. 

The clinical symptoms of Alzheimer’s patients frequently include loss of body weight and muscle strength during the progressive stages of the disease, which are considered as potential risk factors for the diagnosis of AD^50,51^. In particular, motor dysfunction, for example impaired gait and extrapyramidal signs, is accepted as one of the major symptoms of AD in both patients and animal models^52,53^. To ameliorate these negative effects, we previously established an animal model that expresses the *PS2m* transgene in skeletal muscle^42^, and demonstrated the reduction in accumulated Aβ with treadmill exercise^5^. In the present study, we also found that the accumulation of Aβ in skeletal muscle dramatically decreased in transgenic mice for AD (NSE/PS2m) after treadmill exercise (Fig. 2), which is consistent with our previous findings. As described above, chronic exercise training can exert a significant effect on lowing the Aβ burden in the brain, suggesting that regular physical activity may prevent the progression of AD in both the central and peripheral nervous systems. 

Although the molecular mechanism underlying the decline in motor performance has not yet been elucidated, a previous study revealed that the loss of muscle mass in AD patients was positively correlated with brain shrinkage, making it a valid marker of AD and suggesting that the progressive impairments of the brain and skeletal muscle may share a common pathology^54^. Additionally, previous research using APP-overexpressing transgenic mice indicated that the accumulations of Aβ with abnormal APP metabolism in skeletal muscle may lead to altered calcium homeostasis and induce dysregulation of fat and glucose metabolism^43,55^. Therefore, the present study focused on the specific effect of Aβ toxicity in skeletal muscle, and changes in energy metabolism in response to endurance exercise, potentially via the upregulation of several cellular energy sensors (AMPK, ACC, and GLUT4) and a neurotropic factor (BDNF). 

There was a significant increase in the level of p-AMPK, p-ACC, and GLUT4 proteins in skeletal muscle of Tg mice treated with treadmill exercise, in comparison to sedentary Tg mice. Recently, several studies have reported that AD is linked to components of energy metabolism such as mitochondrial dysfunction, defects in glucose uptake, and calcium homeostasis^14,15^, which could potentially be attributed to flawed AMPK expression^16^. AMPK plays a diverse role in energy homeostasis in skeletal muscle^12,13^ as it activates glucose transporters, including GLUT4, for glucose metabolism during muscle contraction^18,19^ and phosphorylates ACC for lipid metabolism^20,21^. Furthermore, AMPK plays a major role in neuroprotection through its downstream effects; most importantly, the inhibition of Aβ production^16^. The phosphorylation of AMPK can decrease Aβ levels in neurons via targeted phosphorylation of ACC^30,31^. Therefore, increased levels of AMPK, ACC, and GLUT4, in response to treadmill exercise, could potentially passively ameliorate or prevent AD pathogenesis through the improvement of muscle energy metabolism. 

BDNF is critical for the survival of neurons and has a crucial role in facilitating learning and memory, which is increased after exercise^56,57^. However, a reduction of BDNF in the hippocampus in relation to the progression of AD has been demonstrated in transgenic mice^58^ and AD patients^59^. In our previous study, we found that hippocampal BDNF was increased by treadmill exercise in NSE/PS2m mice^49^. BDNF is also expressed in skeletal muscle, where it plays an important role in regulating body weight and fat oxidation through the phosphorylation of AMPK and ACC following exercise^26,27^. Taken together, these studies suggest that exercise-induced BDNF upregulation can contribute not only to central nervous system homeostasis, but also to the energy homeostasis in skeletal muscle. The results in the current study demonstrated an increase in the expression of BDNF in skeletal muscle of treadmill-exercised Tg and non-TG mice, indicating that exercise improves fat metabolism by upregulating BDNF levels. Although the mechanism by which exercise upregulated the expression of BDNF is unclear, the data highlight the beneficial effects of physical exercise on BDNF action in both central and peripheral organs. 

Recent findings suggest that molecular regulation of various cellular events, such as fatty acid oxidation and glucose uptake, in skeletal muscle in response to contractile function during exercise^60^. For instance, transgenic mice with muscle-specific inactivation of AMPK showed a decline in muscle glucose metabolism and exercise capacity^61^. In the present study, the protein levels of p-AMPK, p-ACC, BDNF, and GLUT4 were downregulated in Tg mice compared to non-Tg mice (Fig. 3), indicating that AD mice may have poor running ability, similar to the physical inactivity observed in AD patients. However, a limitation in the current study is that both Tg mice and non-Tg mice were made to run on a treadmill at a consistent running speed. In a recent previous study using rats and mice, exercise intensity was defined using the lactate threshold (LT) as a physiologic index during a single bout of running on a rodent treadmill^62,63^. A rat model of type II diabetes further revealed a lower exercise capacity based on the LT, compared to their genetic control subjects^64^. In future studies, we will identify the optimal intensity for exercise regimens in conjunction with the symptoms of AD, which may address such remaining issues for the clinical use of exercise for AD patients. 

In conclusion, our results revealed that chronic treadmill exercise might reduce Aβ deposition and upregulate the protein levels of p-AMPK, p-ACC, BDNF, and GLUT4 in skeletal muscle of Tg mice modeling AD. Our previous work and other studies further suggest that exercise training, such as forced treadmill and voluntary exercise, may represent a potent non-pharmacologic intervention for improving memory deficits associated with AD^38-41^. Taken together, this suggests that regular physical activity may be an effective therapeutic strategy for the reduction and prevention of the pathogenic progression of AD, in non-neuronal organs such as skeletal muscle, as well in the brain. 

## References

[1] Selkoe DJ. (2001). Alzheimer’s disease: genes, proteins, and therapy. *Physiol Rev*.

[2] Gandy S. (2002). Molecular basis for anti-amyloid therapy in the prevention and treatment of Alzheimer’s disease. *NBA*.

[3] Tanzi RE., Bertram L. (2005). Twenty Years of the Alzheimer’s Disease Amyloid Hypothesis: A Genetic Perspective. *Cell*.

[4] Kuo YM., Kokjohn TA., Watson MD., Woods AS., Cotter RJ., Sue LI., Kalback WM., Emmerling MR., Beach TG., Roher AE. (2000). Elevated abeta42 in skeletal muscle of Alzheimer disease patients suggests peripheral alterations of AbetaPP metabolism. *Am J Pathol*.

[5] Cho JY., Hwang DY., Kang TS., Shin DH., Hwang JH., Lim CH., Lee SH, Lim HJ, Min SH, Seo SJ, Song YS, Nam KT, Lee KS, Cho JS, Kim YK (2003). Use of NSE/PS2m-transgenic mice in the study of the protective effect of exercise on Alzheimer’s disease. *J Sports Sci*.

[6] Guérin O., Andrieu S., Schneider SM., Cortes F., Cantet C., Gillette-Guyonnet S., Vellas B. (2009). Characteristics of Alzheimer’s disease patients with a rapid weight loss during a six-year follow-up. *Clin Nutr*.

[7] White H., Pieper C., Schmader K., Fillenbaum G. (1996). Weight change in Alzheimer’s disease. *J Am Geriatr Soc*.

[8] Tamura BK., Masaki KH., Blanchette P. (2007). Weight loss in patients with Alzheimer’s disease. *J Nutr Elder*.

[9] Aggarwal NT., Wilson RS., Beck TL., Bienias JL., Bennett DA. (2006). Motor dysfunction in mild cognitive impairment and the risk of incident Alzheimer disease. *Arch Neurol*.

[10] Mukhamedyarov MA., Grishin SN., Yusupova ER., Zefirov AL., Palotás A. (2009). Alzheimer’s beta-amyloid-induced depolarization of skeletal muscle fibers: implications for motor dysfunctions in dementia. *Cell Physiol Biochem*.

[11] Craft S. (2009). The role of metabolic disorders in Alzheimer disease and vascular dementia: two roads converged. *Arch Neurol*.

[12] Hardie DG. (2008). AMPK: a key regulator of energy balance in the single cell and the whole organism. *Int J Obes*.

[13] Lage R., Diéguez C., Vidal-Puig A., López M. (2008). AMPK: a metabolic gauge regulating whole-body energy homeostasis. *Trends Mol Med*.

[14] Mosconi L., Pupi A., De Leon MJ. (2008). Brain glucose hypometabolism and oxidative stress in preclinical Alzheimer’s disease. *Ann N Y Acad Sci*.

[15] Galindo MF., Ikuta I., Zhu X., Casadesus G., Jordán J. (2010). Mitochondrial biology in Alzheimer’s disease pathogenesis. *J Neurochem*.

[16] Haapasalo A., Soininen H., Hiltunen M. (2011). AMP-activated protein kinase: a potential player in Alzheimer’s disease. *J Neurochem*.

[17] Hayashi T., Hirshman MF., Kurth EJ., Winder WW., Goodyear LJ. (1998). Evidence for 5’ AMP-activated protein kinase mediation of the effect of muscle contraction on glucose transport. *Diabetes*.

[18] Kurth-Kraczek EJ., Hirshman MF., Goodyear LJ., Winder WW. (1999). 5’ AMP-activated protein kinase activation causes GLUT4 translocation in skeletal muscle. *Diabetes*.

[19] Hardie DG. (2011). Energy sensing by the AMP-activated protein kinase and its effects on muscle metabolism. *Proc Nutr Soc*.

[20] Carling D. (2005). AMP-activated protein kinase: balancing the scales. *Biochimie*.

[21] Fediuc S., Gaidhu MP., Ceddia RB. (2006). Regulation of AMP-activated protein kinase and acetyl-CoA carboxylase phosphorylation by palmitate in skeletal muscle cells. *J Lipid Res*.

[22] Ruderman NB., Park H., Kaushik VK., Dean D., Constant S., Prentki M., Saha AK (2003). AMPK as a metabolic switch in rat muscle, liver and adipose tissue after exercise. *Acta Physiol Scand*.

[23] Winder WW., Thomson DM. (2007). Cellular energy sensing and signaling by AMP-activated protein kinase. *Cell Biochem Biophys*.

[24] Lipsky RH., Marini AM. (2007). Brain-derived neurotrophic factor in neuronal survival and behavior-related plasticity. *Ann N Y Acad Sci*.

[25] Laske C., Stransky E., Leyhe T., Eschweiler GW., Wittorf A., Richartz E., Bartels M., Buchkremer G., Schott K. (2006). Stage-dependent BDNF serum concentrations in Alzheimer’s disease. *J Neural Transm*.

[26] Pedersen BK., Pedersen M., Krabbe KS., Bruunsgaard H., Matthews VB., Febbraio MA. (2009). Role of exercise-induced brain-derived neurotrophic factor production in the regulation of energy homeostasis in mammals. *Exp Physioly*.

[27] Matthews VB., Aström M-B., Chan MHS., Bruce CR., Krabbe KS., Prelovsek O., Akerström T., Yfanti C., Broholm C., Mortensen OH., Penkowa M., Hojman P., Zankari A., Watt MJ., Bruunsgaard H., Pedersen BK., Febbraio MA. (2009). Brain-derived neurotrophic factor is produced by skeletal muscle cells in response to contraction and enhances fat oxidation via activation of AMP-activated protein kinase. *Diabetologia*.

[28] Cai Z., Yan LJ., Li K., Quazi SH., Zhao B. (2012). Roles of AMP-activated Protein Kinase in Alzheimer’s Disease. *Neuromol Med*.

[29] Saha A., Coughlan K., Valentine R., Ruderman N. (2014). AMPK activation: a therapeutic target for type 2 diabetes?. *Diabetes Metab Syndr Obes*.

[30] Vingtdeux V., Giliberto L., Zhao H., Chandakkar P., Wu Q., Simon JE., Janle EM., Lobo J., Ferruzzi MG., Davies P., Marambaud P. (2010). AMP-activated protein kinase signaling activation by resveratrol modulates amyloid-beta peptide metabolism. *J Biol Chem*.

[31] Won JS., Im YB., Kim J., Singh AK., Singh I. (2010). Involvement of AMP-activated-protein-kinase (AMPK) in neuronal amyloidogenesis. *Biochem Biophys Res Commun*.

[32] Frontera WR., Meredith CN., O’Reilly KP., Evans WJ. (1990). Strength training and determinants of VO2max in older men. *J Appl Physiol*.

[33] Häkkinen K., Kraemer WJ., Newton RU., Alen M. (2001). Changes in electromyographic activity, muscle fibre and force production characteristics during heavy resistance/power strength training in middle-aged and older men and women. *Acta Physiol Scand*.

[34] Colcombe SJ., Erickson KI., Raz N., Webb AG., Cohen NJ., McAuley E., Kramer AF. (2003). Aerobic fitness reduces brain tissue loss in aging humans. *J Gerontol A Biol Sci Med Sci*.

[35] Ruscheweyh R., Willemer C., Krüger K., Duning T., Warnecke T., Sommer J., Völker K, Ho HV, Mooren F, Knecht S (2011). Physical activity and memory functions: an interventional study. *Neurobiol Aging*.

[36] Santana-Sosa E., Barriopedro MI., López-Mojares LM., Pérez M., Lucia A. (2008). Exercise training is beneficial for Alzheimer’s patients. *Int J Sports Med*.

[37] Rolland Y., Abellan van Kan G., Vellas B. (2008). Physical Activity and Alzheimer’s Disease: From Prevention to Therapeutic Perspectives. *J Am Med Dir Assoc*.

[38] Um HS., Kang EB., Koo JH., Kim HT., Lee J., Kim EJ., Yang CH., An GY., Cho IH., Cho JY. (2011). Treadmill exercise represses neuronal cell death in an aged transgenic mouse model of Alzheimer’s disease. *Neurosci Res*.

[39] Kang EB., Kwon IS., Koo JH., Kim EJ., Kim CH., Lee J., Yang CH., Lee YI., Cho IH., Cho JY. (2013). Treadmill exercise represses neuronal cell death and inflammation during Aβ-induced ER stress by regulating unfolded protein response in aged presenilin 2 mutant mice. *Apoptosis*.

[40] Koo JH., Kang EB., Oh YS., Yang DS., Cho JY. (2016). Treadmill exercise decreases amyloid-β burden possibly via activation of SIRT-1 signaling in a mouse model of Alzheimer’s disease. *Exp Neurol*.

[41] Adlard PA., Perreau VM., Pop V., Cotman CW. (2005). Voluntary exercise decreases amyloid load in a transgenic model of Alzheimer’s disease. *J Neurosci*.

[42] Hwang DY., Chae KR., Kang TS., Hwang JH., Lim CH., Kang HK., Goo JS, Lee MR., Lim HJ., Min SH., Cho JY., Hong JT., Song CW., Paik SG., Cho JS., Kim YK. (2002). Alterations in behavior, amyloid beta-42, caspase-3, and Cox-2 in mutant PS2 transgenic mouse model of Alzheimer’s disease. *FASEB J*.

[43] Shabrokh E., Kavanaugh J., McMillan R., Wu Y., Hulver M., Frisard M. (2015). Substrate Metabolism and Mitochondrial Function in Skeletal Muscle of Amyloid Precursor Protein-Overexpressing Mice. *Ann Clin Lab Res*.

[44] Boncompagni S., Moussa CE., Levy E., Pezone MJ., Lopez JR., Protasi F., Shtifman A. (2012). Mitochondrial dysfunction in skeletal muscle of amyloid precursor protein-overexpressing mice. *J Biol Chem*.

[45] Monteiro-Cardoso V., Castro M., Oliveira MM., Moreira P., Peixoto F., Videira R. (2015). Age-Dependent Biochemical Dysfunction in Skeletal Muscle of Triple- Transgenic Mouse Model of Alzheimer’s disease. *Curr Alzheimer Res*.

[46] Thomson DM., Winder WW. (2009). AMP-activated protein kinase control of fat metabolism in skeletal muscle. *Acta Physiol (Oxf)*.

[47] Zorzano A., Palacin M., Guma A. (2005). Mechanisms regulating GLUT4 glucose transporter expression and glucose transport in skeletal muscle. *Acta Physiol Scand*.

[48] Howlett DR., Jennings KH., Lee DC., Clark MS., Brown F., Wetzel R., Wood SJ., Camilleri P., Roberts GW. (1995). Aggregation state and neurotoxic properties of Alzheimer beta-amyloid peptide. *Neurodegeneration*.

[49] Um HS., Kang EB., Leem YH., Cho I-H., Yang CH., Chae KR., Hwang DY., Cho JY. (2008). Exercise training acts as a therapeutic strategy for reduction of the pathogenic phenotypes for Alzheimer’s disease in an NSE/APPsw-transgenic model. *Int J Mol Med*.

[50] Sergi G., De Rui M., Coin A., Inelmen EM., Manzato E. (2013). Weight loss and Alzheimer’s disease: temporal and aetiologic connections. *Proc Nutr Soc*.

[51] McKhann G., Drachman D., Folstein M., Katzman R., Price D., Stadlan EM. (1984). Clinical diagnosis of Alzheimer’s disease: report of the NINCDS-ADRDA Work Group under the auspices of Department of Health and Human Services Task Force on Alzheimer’s Disease. *Neurology*.

[52] Wirths O., Bayer TA. (2008). Motor impairment in Alzheimer’s disease and transgenic Alzheimer’s disease mouse models. *Genes Brain Behav*.

[53] Buchman AS., Bennett DA. (2011). Loss of motor function in preclinical Alzheimer’s disease. *Expert Rev Neurother*.

[54] Burns JM., Johnson DK., Watts A., Swerdlow RH., Brooks WM. (2010). Reduced lean mass in early Alzheimer disease and its association with brain atrophy. *Arch Neurol*.

[55] Moussa CE., Fu Q., Kumar P., Shtifman A., Lopez JR., Allen PD., LaFerla F., Weinberg D., Magrane J., Aprahamian T., Walsh K., Rose KM., Querfurth HW. (2006). Transgenic expression of beta-APP in fast-twitch skeletal muscle leads to calcium dyshomeostasis and IBM-like pathology. *FASEB J*.

[56] Lee MC., Okamoto M., Liu YF., Inoue K., Matsui T., Nogami H. (2012). Voluntary resistance running with short distance enhances spatial memory related to hippocampal BDNF signaling. *J Appl Physiol*.

[57] Vaynman S., Ying Z., Gomez-Pinilla F. (2004). Hippocampal BDNF mediates the efficacy of exercise on synaptic plasticity and cognition. *Eur J Neurosci*.

[58] Peng S., Garzon DJ., Marchese M., Klein W., Ginsberg SD., Francis BM., Mount HT., Mufson EJ., Salehi A., Fahnestock M. (2009). Decreased brain-derived neurotrophic factor depends on amyloid aggregation state in transgenic mouse models of Alzheimer’s disease. *J Neurosci*.

[59] Connor B., Young D., Yan Q., Faull RLM., Synek B., Dragunow M. (1997). Brain-derived neurotrophic factor is reduced in Alzheimer’s disease. *Brain Res Mol Brain Res*.

[60] Richter EA., Ruderman NB. (2009). AMPK and the biochemistry of exercise: implications for human health and disease. *Biochem J*.

[61] Fujii N., Seifert MM., Kane EM., Peter LE., Ho RC., Winstead S., Hirshman MF., Goodyear LJ. (2007). Role of AMP-activated protein kinase in exercise capacity, whole body glucose homeostasis, and glucose transport in skeletal muscle -insight from analysis of a transgenic mouse model. *Diabetes Res Clin Pract*.

[62] Soya H., Mukai A., Deocaris CC., Ohiwa N., Chang H., Nishijima T., Fujikawa T., Togashi K., Saito T. (2007). Threshold-like pattern of neuronal activation in the hypothalamus during treadmill running: Establishment of a minimum running stress (MRS) rat model. *Neurosci Res*.

[63] Inoue K., Okamoto M., Shibato J., Lee MC., Matsui T., Rakwal R., Soya H. (2015). Long-Term Mild, rather than Intense, Exercise Enhances Adult Hippocampal Neurogenesis and Greatly Changes the Transcriptomic Profile of the Hippocampus. *PLoS ONE*.

[64] Shima T., Matsui T., Jesmin S., Okamoto M., Soya M., Inoue K., Liu YF., Torres-Aleman I. (2017). Moderate exercise ameliorates dysregulated hippocampal glycometabolism and memory function in a rat model of type 2 diabetes. *Diadetologia*.

